# Clinical Significance of A Single Multi-Slice CT Assessment in Patients with Coronary Chronic Total Occlusion Lesions Prior to Revascularization

**DOI:** 10.1371/journal.pone.0098242

**Published:** 2014-06-06

**Authors:** Xinkai Qu, Weiyi Fang, Kaizheng Gong, Jianding Ye, Shaofeng Guan, Ruogu Li, Yingjia Xu, Yan Shen, Min Zhang, Hua Liu, Wenhui Xie

**Affiliations:** 1 Department of Cardiology, Shanghai Chest Hospital, Shanghai Jiao Tong University, Shanghai, China; 2 Department of Cardiology, The Second Clinical Medical School of Yangzhou University, Jiangsu Province, China; 3 Department of Radiology, Shanghai Chest Hospital, Shanghai Jiao Tong University, Shanghai, China; 4 Department of Nuclear medicine, Shanghai Chest Hospital, Shanghai Jiao Tong University, Shanghai, China; University of Groningen, Netherlands

## Abstract

Accurate assessment of coronary chronic total occlusion (CTO) lesion is essential to design an appropriate procedural strategy before revascularization. The present study aims to evaluate the significance of a single multislice computed tomography (MSCT) examination in patients with CTO lesion. We retrospectively analyzed the clinical data of 23 CTO lesions in twenty patients underwent computed tomography coronary angiography (CTCA) and SPECT. The CTCA was more powerful and sensitive to determine the CTO lesion length (100% v.s 47.8%) and to identify the length and location of calcification in occluded vessels compared with the coronary angiography (CAG). The LVEF measured by MSCT was comparable to that from the gated SPECT. Myocardial perfusion imaging showed that the location of the early defect region identified by MSCT was corresponded to the nuclide filling defect on the stressed ^201^thallium-SPECT imaging. The late hyperenhancement on MSCT was presented as incomplete nuclide filling on the ^99m^Tc-MIBI imaging. The results suggested that a single MSCT examination in previous myocardial infarction without revascularization facilitates to provide some valuable information on the nature of the occluded lesion, myocardial perfusion and globe cardiac function, which would be helpful to design appropriate revascularization strategy in these subjects.

## Introduction

Chronic total occlusion (CTO) of a coronary artery was documented in nearly 15% patients undergoing coronary angiography (CAG), and represents of 10% lesions treated by percutaneous coronary intervention (PCI)[1]. Successful recanalization of CTO lesions in patients with viable myocardium not only facilitates to reduce angina symptoms, avoid bypass surgery and decrease incidence of myocardial infarction, but also may improve long-term survival[2], [3]. An analysis of 25 years of experience in the Mayo Clinic showed that the procedural success rate for CTO remained around 70%[4]. However, with the remarkable advancements in the instruments and the techniques of PCI for CTO lesions, the success rate of PCI for CTO in the few international cardiovascular interventional centers have raised up to 80% to 90%[5]. It is noted that the application of new instruments and the techniques of PCI often are limited due to various reasons. Therefore, the recanalization of CTO lesion still remains as one of major challenges for the most interventional cardiologists in current clinical practice. Using the traditional antegrade wire approach, the lesion characteristics including long lesion length and occlusion duration, no stump, presence of a large side branch with a takeoff adjacent to the occlusion, serious calcification and bad collateral circulation, often became the unfavorable factors for successful recanalization for CTO lesion[6], [7]. Therefore, a detailed comprehensive assessment for CTO arteries has become very important before PCI revascularization.

Recently, noninvasive imaging techniques, including computed tomography coronary angiography (CTCA), magnetic resonance imaging and single-photon emission computed tomography (SPECT), on the assessment of coronary artery disease have been developed and well documented in many studies[8]–[10]. For example, based on its high spatial and temporal resolution, multislice computed tomography (MSCT) can not only non-invasively assess the coronary arteries and detect coronary stenosis (≥50%) with high sensitivity and specificity compared with quantitative CAG, but also provides excellent information concerning cardiac function and myocardial perfusion[11], [12]. More recently, Schuleri *et al* [13] demonstrated that a delayed enhancement (DE)-MSCT had ability to provide a more detailed assessment of the peri-infarct zone in a model of pig with chronic myocardial infarction compared with magnetic resonance imaging. However, few data are available on the value of MSCT in patients with CTO[14]. Also, it is noted that the diagnosis of CTO by MSCT sometimes may be underestimated or missed because the vessel distal to the CTO is often visualized or opacified with contrast because of retrograde collateral flow[15], [16]. Therefore, we hypothesized that combination of multiple non-invasive modalities can provide more accurate information in the assessment of CTO lesion compared with the traditional CAG. The aim of this study was to evaluate the diagnostic value of a single MSCT examination on patients with CTO lesion by retrospectively comparing the data from MSCT, SPECT imaging and CAG.

## Methods

The research was approved by Shanghai Chest Hospital ethics committee. Written consent was given by the patients for their information to be stored in the hospital database and used for research.

### Patients

During Jan 2010 to Dec 2010, a total of 600 patients with coronary heart disease were diagnosed in the Shanghai Chest Hospital Affiliated to Shanghai Jiaotong University. Patients with atrial fibrillation, renal insufficiency (serum creatinine >120 mmol/L), known allergy to iodine contrast media, unstable clinical conditions, and other routine contraindications for MSCT or SPECT were excluded. After exclusion, 20 patients with a total of 23 CTO lesions identified by invasive CAG and MSCT were entered in this retrospective study.

### MSCT Data Acquisition

MSCT imaging was performed using a 64-detector single-source CT scanner (Philips Medical Systems Corporation). Beta-blockade was used if the heart rate was >65 beats/min. Scanning parameters were as follows: tube voltage 80 to 100 kV, tube current 650–800 mA, depending on body mass index, slice thickness  = 0.6 mm, increment  = 0.3 mm. An 80 ml bolus of Ultravist 370 was injected intravenously at a rate of 4 ml/s, followed by 25 ml saline flush at same flow rate was used. To time the scan, automated detection of peak enhancement in the aortic root was used. All images were acquired during an inspiratory breath hold, while an electrocardiogram (ECG) was used simultaneously for retrospective analysis of the data. MSCT images were acquired at 45 seconds and 5 minutes after bolus injection, and raw data were stored for subsequent reconstruction. Source images were analyzed by software, at 60–75% of the RR interval with a retrospective multi-cycle ECG gating algorithm, slice thickness  = 3 mm, reconstruction increment  = 0.3 mm to assure image quality. Axial slices were reconstructed for assessment of necrotic myocardium, various myocardial signal intensities, left ventricular ejection fraction (LVEF) and coronary artery stenosis. Radiation dose was determined based on the dose-length product documented in the CT scan protocol, separately for the ‘test bolus’ acquisition and the coronary CTA acquisition. Effective dose was estimated based on the dose-length product, using a conversion factor of 0.014 for chest CT in adults.

### MSCT Data Analysis

Images were analyzed by two experienced cardiovascular radiologists, using a dedicated workstation with multiplanar reconstruction images, and volume rendering (VR). The VR allowed reconstruction of three-dimensional (3D) images (Tree, Heart, Outline). For each slice, endocardial and epicardial contours of the left ventricle in both end-systolic and end-diastolic periods were drawn manually and independently by a radiologist, and global function parameters, including end-systolic volume, end-diastolic volume and LVEF were calculated. To assess myocardial viability, the signal intensity in a separate myocardial area was measured.

### Thallium-201 Chloride (^201^TICI) Perfusion imaging After Dobutamine Stress

Dobutamine was injected intravenously at a rate of 5 µg/kg/min initially, then increased by 5 µg/kg/min every 3 minutes to 20 µg/kg/min. Injection of 111–148 MBq (3–4 mCi) ^201^TICI was performed when stress was induced, as indicated by any of the following: heart rate 85% of the expected highest heart rate or ≥130 bpm; severe angina symptoms; serious arrhythmia; systolic blood pressure ≥28 KPa or blood pressure decreased by >3 KPa; ST segment decreased by ≥2 mm. Myocardium perfusion imaging was performed 10 minutes later, and nuclide myocardial perfusion was observed.

### Delayed ^201^TICI Resting Perfusion imaging

Delayed ^201^TICI resting perfusion imaging was performed 4 hours after the first examination, and ^201^TI distribution was observed. ^201^TI redistribution was defined as an ischemic myocardial region; no redistribution was defined as an infarcted region; and reverse redistribution was defined as a damaged region.

### Technetium-99m Hexakis-2-methoxy-2-isobutylisonitrile (^99m^Tc-MIBI) Perfusion Imaging

Nitroglycerin (0.5 mg) was given immediately after delayed ^201^TICI resting perfusion imaging. ^99m^Tc-MIBI 1110 MBq (30 mCi) was injected after 5 minutes, followed by ^99m^Tc-MIBI perfusion imaging 1 hour later. The region with residual nuclide filling defect was defined as necrotic myocardium.

All above SPECT data were collected at three examination time-points. Parameter settings were as follows: low energy high resolution parallel-hole collimators, 64×64 matrix, 1.00 zoom, peak energy 70–80 keV, window width 20%, and dual-detector with an angle of 60°. Original data were collected for reconstruction. Horizontal long-axis, vertical long-axis and short-axis images were acquired to define early perfusion defect (ED) regions and LVEF, and to compare qualitatively between MSCT-derived zones.

### Invasive CAG Examination

After the above noninvasive imaging examination, a conventional CAG was performed in all 20 patients according to the standard technique. An experienced operator blinded to the MSCT data visually evaluated the coronary angiograms.

### Statistical Analysis

Continuous variables were expressed as mean ± standard deviation. Agreement between MSCT- and SPECT-derived LVEF was assessed with the Pearson's correlation coefficient and by Bland-Altman analysis.25 Statistical analysis was performed with SPSS 13.0 software. For Bland-Altman analysis, MedCalc® software was used. Statistical significance was defined as two-sided P value <0.05.

## Results

### Clinical characteristics

The study population consisted of seventeen men and three women, mean age was 66±10 years. Mean heart rate was 61±3 beats/min, duration of breath hold was 15s. Clinical characteristics of the study population are summarized in Table 1. All patients underwent coronary artery MSCT and invasive CAG examination. Effective radiation dose for dual-stage MSCT scanning was 31.60±1.92 mGy. Among, 10 patients also performed successfully myocardial perfusion SPECT imaging. Measurement of serum creatinine remained similar level before and after there invasive examines. Subsequently, successful revascularization procedure were performed in 17/23 CTO lesions according the information from MSCT examination.

**Table 1:** Clinical characteristics of the study population (*n* = 20).

Mean age (yrs)	66±10
Male, n (%)	17 (85.0%)
Smoker	4 (20.0%)
Diabetes mellitus	4 (20.0%)
Hypertension	12 (60.0%)
Previous infarction	5 (25.0%)

### Comparative of CTO lesion features: CTCA vs. CAG

**Figure 1:**
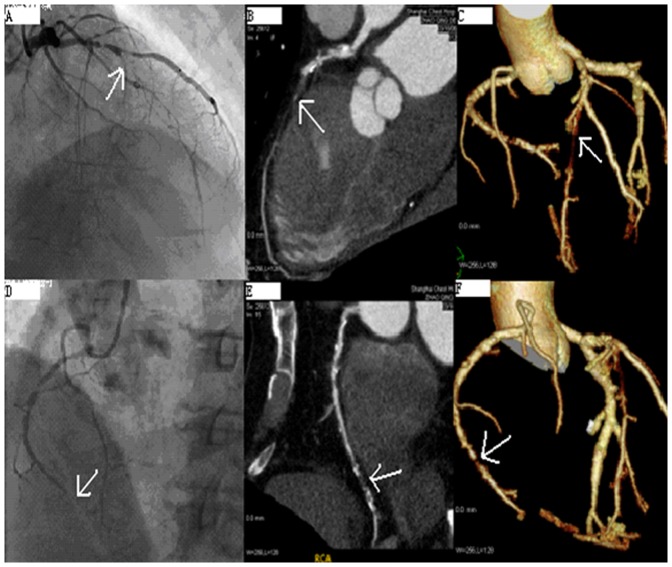
Representative Reconstructed Images of CTO Lesions at the Left Anterior Descending Coronary Artery (LAD) and Right Coronary Artery (RCA). 1A, 1D: Coronary angiography (CAG) image; 1B, 1E: Multiplanar reconstruction images; 1C, 1F: Three-dimensional volume rendering (Tree) image.

A total of twenty-three CTO lesions were assessed by CTCA and CAG in 20 patients, including eight CTOs in the left anterior descending coronary artery, seven in the left circumflex coronary artery and eight in the right coronary artery. The length of the occluded segment was determined by CTCA examination in all 23 CTOs. In contrast, only 11 CTOs was measured by CAG because of missing collateral angiography. In eleven patients with data acquired both by MSCT and CAG, the length of the CTOs showed no significant difference between two methods (23±12 mm vs. 22±11 mm, P>0.05). However, CTCA had higher sensitivity in the rate of detection of calcification lesion compared with CAG (69.6% vs. 43.4%, P<0.05). Also, the length of calcification lesion could be successfully measured by CTCA (10.1±5.5 mm), but not by CAG. It is noted that CTCA could detect the detailed location (proximal, middle and distal segment) of calcification in occlusive vessels, and facilitated to visualize the tract of the occluded segment by using 3D reconstruction (Fig. 1).

### Comparison of LV function: MSCT vs. SPECT

**Figure 2:**
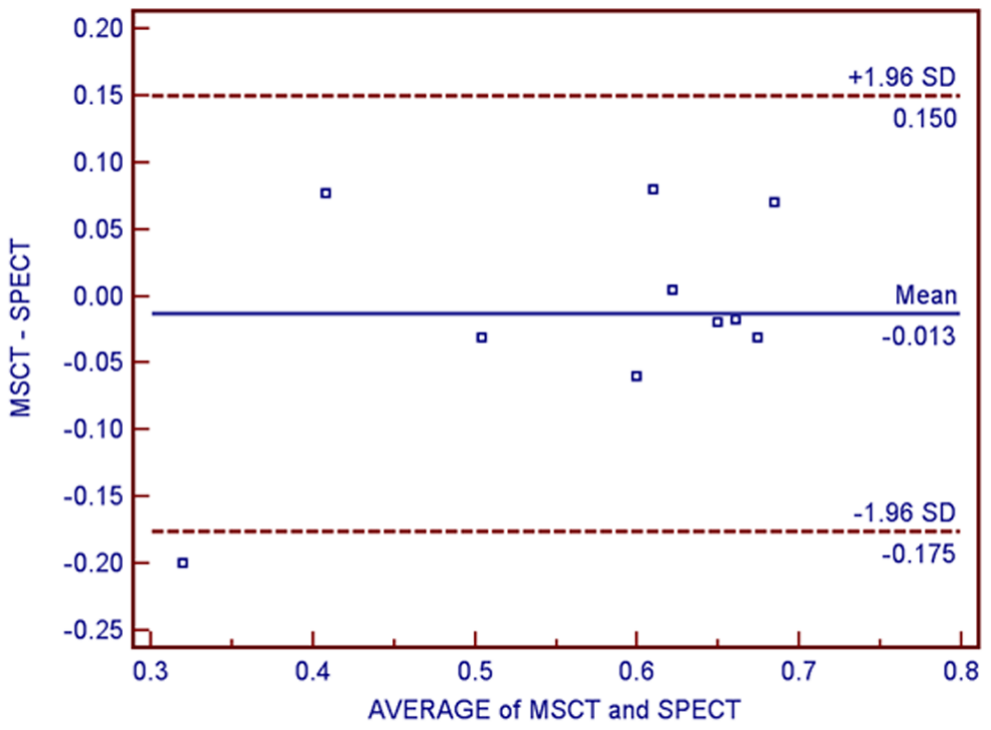
Bland-Altman Plots of Left Ventricular Ejection Fraction using Multislice Computed Tomography (MSCT) and Single-photon Emission Computed Tomography (SPECT).

The assessment of global LV function has important diagnostic and prognostic values in patients with CAD, especially for CTO. The average LVEF (58%±11%) obtained by gated SPECT was comparable to that measured with MSCT (57%±15%). The measurement of LVEF by using MSCT and SPECT methods are highly correlated with a r Pearson coefficient value of 0.79 (P<0.01). Bland-Altman plot analysis indicated a good agreement between the two methods (Fig. 2).

### Comparison of Myocardium Perfusion: MSCT vs. SPECT

Quantitative analysis from MSCT biphasic imaging in all CTO patients showed a statistically significant difference in attenuation values between the normal and the perfusion defect regions in early scanning images (92±13 HU vs. 42±12 HU, P<0.001). Three patients had residual perfusion defect regions in late scanning images and the attenuation values were lower than those in the normal regions (57±16 HU vs. 86±18 HU, P<0.01). Late hyperenhancement (LH) regions were detected in eight patients and had higher attenuation values compared with the normal regions (135±29 HU vs. 86±18 HU, P<0.001).

**Figure 3:**
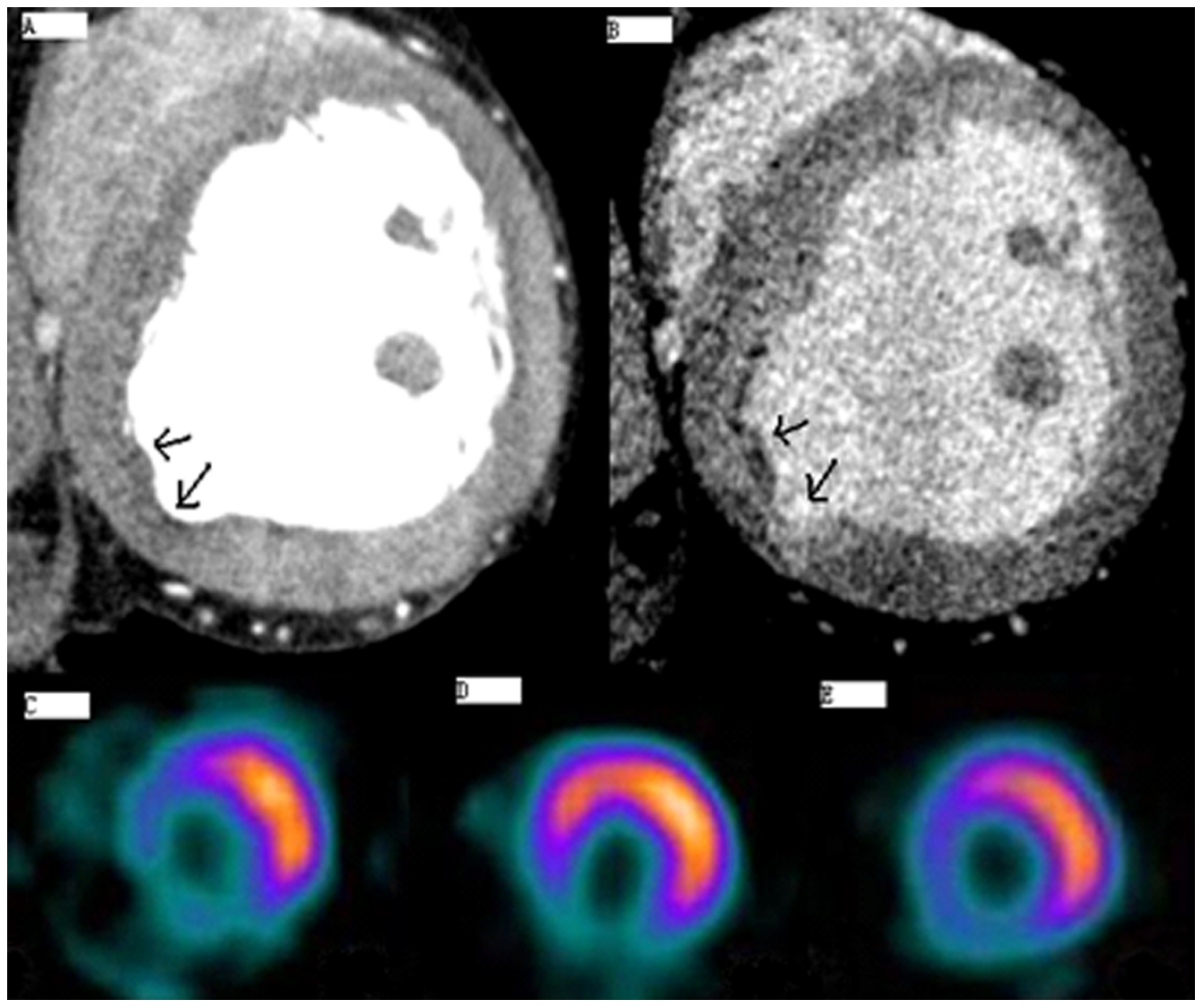
Representative Myocardial Perfusion Images of the Left Anterior Descending Coronary Artery (LAD) and Right Coronary Artery (RCA) using both MSCT and SPECT Examination. 3A: MSCT early scanning; 3B: MSCT late scanning; 3C: Stressed thallium-201 (^201^TI)-SPECT imaging; 3D: ^99m^Tc-MIBI imaging; 3E: ^201^TI-SPECT late resting image.

In addition, table 2 showed that a total of 10 patients with CTO finished myocardial perfusion imaging using both MSCT and SPECT. The location of the early perfusion defect region identified by MSCT was corresponded to the nuclide filling defect detected by stressed ^201^TI-SPECT perfusion imaging. In patients with late hyperenhancement in MSCT, ^99m^Tc-MIBI imaging showed incomplete nuclide filling. By contrast, the complete nuclide filling was shown on ^99m^Tc-MIBI imaging in patients without late hyperenhancement on MSCT scan. Figure 3 shows an example of a large perfusion defect on MSCT and SPECT.

**Table 2:** Myocardial perfusion comparison between MSCT and SPECT.

Patient	CTO location	MSCT			SPECT		
		ED	RD	LH	Stressed image	Delayed resting image	^99m^Tc-MIBI image
1	LADLCX	Anteroseptal	Anteroseptal	Anteroseptal	Anteroseptal, Inferior	INF	INF
2	RCA	Inferior	N	Inferior	Inferior	INF	INF
3	LAD	Anteroseptal	N	Anteroseptal	Anteroseptal	INF	INF
4	RCA	Inferior	N	Inferior	Inferolateral	INF	INF
5	LAD, RCA	Anteroseptal	N	Anteroseptal	Apical`Inferior	INF	CNF at apical wallINF at inferior wall
6	LCX	Anterolateral	N	N	Lateral	INF	CNF
7	LAD	Anteroseptal	N	Anteroseptal	Anteroseptal	INF	INF
8	LAD	Anterior	N	N	Anteroseptal	INF	CNF
9	RCA	Inferior	N	N	Inferior	INF	CNF
10	LAD	Anteroseptal	N	Anteroseptal	Anteroseptal	INF	INF

CTO, chronic total occlusion; ED, early perfusion defect; RD, residual perfusion defect; LH, late hyperenhancement; INF, incomplete nuclide filling; CNF, complete nuclide filling; LCX, left circumflex coronary artery; ^99m^Tc-MIBI, technetium-99m hexakis-2-methoxy-2-isobutylisonitrile.

## Discussion

Accurate assessment of CTO lesion is essential to design the strategy of revascularization before PCI procedure. Accumulating evidence demonstrated that combination of multiple coronary artery imaging techniques provides a comprehensive assessment on coronary artery lesions[17], [18]. In the present study, we evaluate the diagnosis value of a single MSCT examination on CTO lesion by comparative analysis of the data from traditional invasive CAG and myocardial perfusion SPECT imaging.

Many studies have demonstrated that the success rates of PCI for CTO are influenced by marked lesion calcification at the stump, severe tortuosity of the proximal vessel, the orientation and long length of the occluded segment as well as the location of the vessel distal to the occlusion[5], [7]. Our results demonstrated that all above-mentioned unfavorable features of PCI treatment can be easily identified by CTCA. By contrast, the lesion features can not be often well detected by traditional CAG. As a result of the relatively long time that transpires between intravenous injection of contrast and the time required for scan acquisition, the distal site of the occlusion could be identified by retrograde collateral flow of contrast.

Serious calcification was shown to have strong association with the success rate of CTO recanalization[7], [19]. Although sometimes it is difficult to identify calcification using CAG, but CTCA has been confirmed to be a useful tool for the identification of calcification[20]. The present study showed that the location, extent and length of calcification could be accurately evaluated by CTCA.

The most common reasons for procedural failure of PCI for CTOs include the inability to cross the lesion into the true lumen of the distal vessel with a guidewire (>60%), intimal dissection with creation of a false lumen, contrast extravasation, failure to cross the lesion with a balloon, or failure to dilate adequately. In fact, the absence of morphological information of occluded artery including tortuosity or bending, exact occlusion length or vessel diameter, localization of soft or calcified plaque with traditional CAG, significantly increase the difficulty of CTO recanalization by PCI[21]. Therefore, if the features of CTO could be well identified prior to PCI, adequate pre-procedural planning and scheduling could be undertaken. Our results have demonstrated that the features of CTO, including length of occluded segment, location and extent of calcification, orientation and path of the CTO, could be identified by CTCA, suggesting a role of CTCA in assessing CTO features. Also, 3-D vascular reconstruction images for CTO lesion can be used to map and determinate the optimal working view angle that best demonstrates the target lesion with least foreshortening. The information will be helpful to predict the success rate of CTO recanalization and to design optional procedural strategies and guide the operation of PCI[16], [22].

Previous studies have shown that LVEF measured by MSCT had an excellent correlation with that by SPECT or transthoracic echocardiography[12], [23]. Similar with these results, we also showed a good correlation between the data from MSCT and SPECT.

In addition, with its high spatial and temporal resolution achievable, MSCT recently was used to assess cardiac function and myocardial perfusion[11], [12]. Francone et al[24] reported that MSCT could detect 25 of 30 myocardial infarction, showing an overall sensitivity of 83% and specificity of 91%. Henneman et al[25] has demonstrated that MSCT not only was used to detect healed myocardial infarction, evaluate LV function and myocardial perfusion in patients with previous MI, but also showed a good correlation with the results from SPECT examination. However, since the results were from post-myocardial infarction patients, it remains unclear whether the findings can be extended to all patients with a CTO lesion. Delayed hyperenhancement in a chronic collagenous myocardial scar results from an accumulation of contrast media in the interstitial space between collagen fibers, resulting in an increased volume of contrast distribution in the scar tissue compared with that of tightly packed myocytes[26]. The present study showed that the location of the early perfusion defect region identified by MSCT was corresponded to the nuclide filling defect on stressed ^201^TI-SPECT perfusion imaging. The late hyperenhancement on MSCT examination was shown to be incomplete nuclide filling on ^99m^Tc-MIBI imaging. The results suggested that during a single examination, MSCT can provide a comprehensive cardiac assessment of lesion morphological, myocardial perfusion and cardiac function, indicating the feasibility of MSCT in assessment of CTO lesion.

For patients with CTO, comprehensive cardiac assessment, including the occluded segment, cardiac function and the viable myocardium should be performed prior to decision-making. The present study confirmed the ability of MSCT to provide comprehensive cardiac information of CTO lesions. However, there are still some limitations. For example, because of exposure of patients to X-rays and potentially toxic contrast agents, MSCT perfusion imaging is presently unlikely to become a first-line test to assess myocardial viability in all patients. Until now, this modality has only been recommended for use in patients with definite CTO or previous myocardial infarction. Also, it is still difficult and time-consuming to analyze the myocardial perfusion images. With the development of dedicated software, the problem of the heavy workload could be diminished in the future.

## Conclusions

In conclusion, in this study, we have demonstrated that except for screening diagnostic purpose on coronary heart disease, a single MSCT examination in previous myocardial infarction without revascularization might provide some valuable information on the nature of CTO lesion, myocardial perfusion and globe cardiac function, which would be helpful for subsequently designing appropriate revascularization strategy in these subjects.
